# What are the factors affecting the recovery rate of bronchoalveolar lavage fluid?

**DOI:** 10.1111/crj.13462

**Published:** 2021-12-06

**Authors:** Kohei Shikano, Mitsuhiro Abe, Yuki Shiko, Kenji Tsushima, Keiichiro Yoshioka, Tsukasa Ishiwata, Takeshi Kawasaki, Jun Ikari, Jiro Terada, Yohei Kawasaki, Koichiro Tatsumi

**Affiliations:** ^1^ Department of Respirology, Graduate School of Medicine Chiba University Chiba Japan; ^2^ Biostatistics Section, Clinical Research Center Chiba University Hospital Chiba Japan; ^3^ Department of Pulmonary Medicine, School of Medicine International University of Health and Welfare Chiba Japan

**Keywords:** bronchoalveolar lavage, bronchoscopy, interstitial lung disease, lung function, spirometry

## Abstract

**Background:**

Bronchoalveolar lavage (BAL) is a useful examination for the evaluation of interstitial lung disease. A high BAL fluid (BALF) recovery rate is desirable because low recovery rates lead to inaccurate diagnoses and increased adverse events. Few studies have explored whether BALF recovery rates are influenced by clinical factors.

**Objectives:**

This study aimed to identify the clinical parameters affecting the recovery rates of BALF and the extent of their effects.

**Method:**

Data from patients who underwent BAL at the Chiba University Hospital between 2013 and 2019 were retrospectively reviewed. BAL was performed with three aliquots of 50‐ml physiological saline. The potential association of the BALF recovery rate with clinical parameters such as age, sex, smoking status, underlying disease, bronchus used for the procedure and pulmonary function, was analysed.

**Results:**

Eight hundred twenty‐six patients had undergone BAL. The average recovery rate was 52.4%. Factors affecting BALF recovery rates included male sex (odds ratio [OR]: 0.32, 95% confidence interval [CI]: 0.20–0.53, *p* < 0.001); age ≥ 65 years (OR: 0.50, 95% CI: 0.33–0.76, *p* < 0.001); use of the left bronchus (OR: 0.46, 95% CI: 0.30–0.71, *p* = 0.001) and bronchi other than the middle lobe bronchus or lingula (OR: 0.41, 95% CI: 0.25–0.65, *p* < 0.001); and forced expiratory volume in 1 s divided by forced vital capacity <80% (OR: 0.42, 95% CI: 0.40–1.00, *p* < 0.001).

**Conclusion:**

Sex, age, bronchus used for the procedure and pulmonary function may be useful as pre‐procedural predictors of BALF recovery rates.

## 1 INTRODUCTION

Bronchoalveolar lavage (BAL) is useful for the evaluation of interstitial lung disease; it is widely performed because of the information it provides regarding the immune status of the lung.^1^ The cellular analysis of BAL fluid (BALF) can play an essential role in the diagnosis of interstitial lung disease, which is unlike the usual interstitial pneumonia pattern observed on high‐resolution computed tomography (HRCT).^2^, ^3^ Furthermore, BALF neutrophil counts predict early mortality in idiopathic pulmonary fibrosis.^4^ BAL also plays an important role in the diagnosis of infections, such as pneumocystis pneumonia,^5^ pulmonary tuberculosis^6^ and invasive pulmonary aspergillosis,^7^ especially in immunocompromised patients.

It has been suggested that the BALF recovery rate should be more than 30% for an effective diagnosis.^8^ In order to recover sufficient BALF, the bronchoscope should be wedged into position by advancing it into a subsegmental bronchus and occluding the lumen.^9^ If it is not positioned properly, fluid leak around the bronchoscope occurs, leading to low recovery rates. However, even if the procedure is performed correctly, low recovery rates can be observed. Previous studies have reported that age, coughing, smoking history and pulmonary function reduce BALF recovery rates^8^, ^10^, ^11^, ^12^; however, few studies have analysed the extent of the effect that clinical parameters have on BALF recovery rates. This study aimed to assess the factors that affect the recovery rates of BALF, as well as the extent of their effects.

## 2 MATERIALS AND METHODS

### 2.1 Study design and patients

We conducted a retrospective chart review of patients who had undergone BAL at the Chiba University Hospital between January 2013 and August 2019. Patients under 18 years of age, or with no recorded BAL recovery volume, were excluded. All analyses were performed in accordance with the amended Declaration of Helsinki. Written informed consent for bronchoscopy was obtained from each patient. Data anonymization and privacy issues were strictly addressed, and the study protocol (with an opt‐out consent method) was approved by the Human Ethics Committee of our institution (approval number 3833).

### 2.2 BAL procedure

All examinations were performed using a flexible bronchoscope, which was inserted orally under mild sedation following pharyngeal anaesthesia. We administered midazolam as the sedative, pethidine as the analgesic or both intravenously in a routine manner, and additional drugs were administered as needed after the bronchoscope was inserted through the vocal cord. The choice of drugs and their doses were made as deemed appropriate by the bronchoscopist. BAL was performed with three aliquots of 50‐ml physiological saline at room temperature; using a method common in Japan, the saline was gradually injected and then gently suctioned back. The site of BAL was determined based on HRCT findings. The middle lobe bronchus or the lingula was mainly chosen. However, in cases where HRCT showed a lesion in other areas, these were preferred for performing BAL.

### 2.3 Pulmonary function tests

Pulmonary function tests were performed before bronchoscopy in most cases. Vital capacity/predicted percentage for vital capacity (%VC) and forced expiratory volume in 1 s/forced vital capacity (FEV_1_/FVC) were recorded and analysed. Pulmonary function data were not adopted in patients whose disease state was worsened rapidly between pulmonary function tests and bronchoscopy.

### 2.4 HRCT findings

HRCT findings were recorded in the chronic fibrotic interstitial pneumonia (CFIP) group. Each HRCT findings was judged based on the report of David et al,^13^ while discussing with the two pulmonologists, after being independently reviewed by the pulmonologists. The definition of findings was as follows: consolidation: homogeneous increase in pulmonary parenchymal attenuation; ground glass shadow: hazy increased opacity of lung with preservation of bronchial and vascular margins; honeycomb: clustered cystic air spaces, typically of comparable diameters on the order of 3–10 mm but occasionally as large as 2.5 cm; reticulation: collection of innumerable small linear opacities that produce an appearance resembling a net; and traction bronchiectasis: irregular bronchial and bronchiolar dilatation caused by surrounding retractile pulmonary fibrosis.

### 2.5 Statistical analysis

The mean or median of continuous data (depending on their distribution) and range were calculated. Counts and percentages were determined for categorical data. The Mann–Whitney *U* test or one‐way analysis of variance was used to compare the means (or medians). The cut‐off value for good recovery was determined by the average recovery rate. A receiver operating characteristic (ROC) curve analysis was performed to obtain the parameters that affected the recovery rate of BALF. Univariate and multivariate logistic regression analyses were used to identify factors independently affecting the recovery rate of BALF. The independent variables included in the analysis were age, sex, smoking status, disease, use of the bronchus for the procedure and pulmonary function. The same analysis was performed on patients with CFIP. Discrimination through the multivariate logistic regression was assessed using C‐statistics. Two‐sided *p*‐values of <0.05 were considered statistically significant, and all analyses were performed with SAS software v.9.4 for Windows (SAS Institute Inc., Cary, NC, USA) and JMP Pro 13.2.0 software (SAS Institute Inc. Cary, NC, USA).

## 3 RESULTS

### 3.1 Study population and characteristics

**FIGURE 1:**
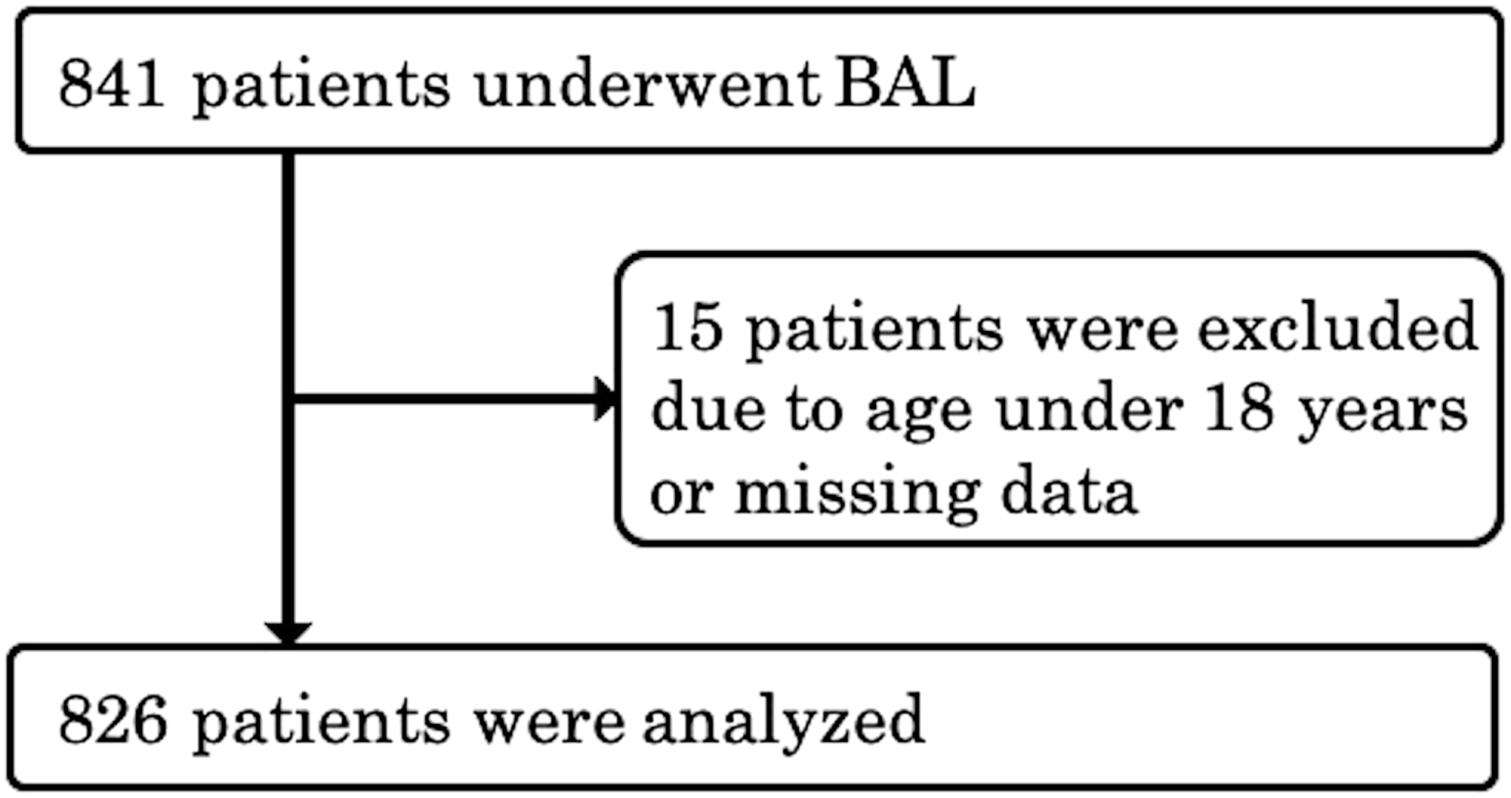
Flow diagram for study participants

In total, 841 patients underwent BAL; 15 patients were excluded according to the criteria (Figure 1). Characteristics of the 826 included patients are summarized in Table 1. There were 476 (57.6%) males and 350 (42.4%) females, with a median age of 67 years (range, 19–89 years). CFIP without acute exacerbation (AE) was the most common diagnosis (*n* = 246 cases, 29.8%), followed by sarcoidosis and AE of CFIP/acute respiratory distress syndrome (ARDS). Pulmonary function tests were performed in 546 patients (66.1%). The relationship between the site performed BAL and HRCT findings in cases underwent pulmonary function tests are shown in Table S1.

**TABLE 1:** Clinical characteristics of the 826 patients

Characteristics		Number of patients (*n*)	
Total		826	
Age, years	Median (range)	67	19–89
Sex, *n*	Male	476	57.6%
	Female	350	42.4%
Smoking status, *n*	Non‐smoker	321	40.0%
	Ex/current smoker	481	60.0%
Final diagnosis, *n*	CFIP without AE	246	29.8%
	Sarcoidosis	187	22.6%
	AE of CFIP/ARDS	36	4.4%
	Others	357	43.2%
Pulmonary function tests, *n*	%VC ≥ 65%	419	76.5%
	FEV1/FVC ≥ 80%	328	59.9%

Abbreviations: AE, acute exacerbation; ARDS, acute respiratory distress syndrome; CFIP, chronic fibrotic interstitial pneumonia; FEV_1_/FVC, forced expiratory volume in 1 s divided by forced vital capacity; VC, vital capacity.

### 3.2 The cut‐off points for %VC and FEV_1_/FVC

**FIGURE 2:**
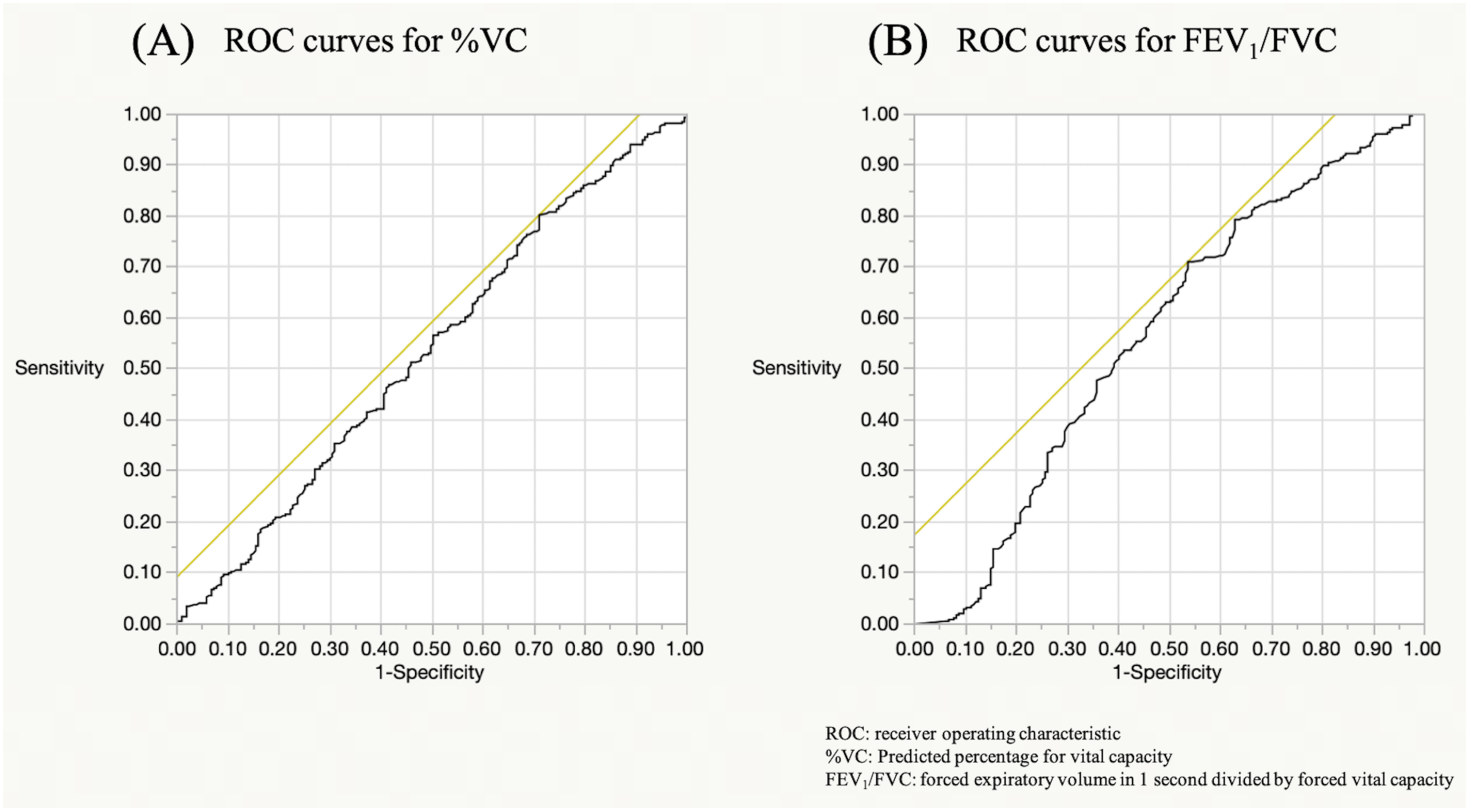
ROC curves for %VC and FEV_1_/FVC, depending on whether the recovery rate was 50% or more. (a) ROC curves for %VC; AUC 0.53; cut‐off point for %VC was calculated as 64.8%. (b) ROC curves for FEV_1_/FVC; AUC 0.57; cut‐off point for FEV_1_/FVC was calculated as 78.9%. %VC, predicted percentage for vital capacity; AUC, area under the curve; FEV_1_/FVC, forced expiratory volume in 1 s divided by forced vital capacity; ROC, receiver operating characteristic

Figure 2 shows the ROC curves used to calculate the cut‐off values for %VC and FEV_1_/FVC; an area under the curve (AUC) analysis was conducted, considering a recovery rate of 50% or more. Cut‐off points for %VC and FEV_1_/FVC were calculated as 64.8% and 78.9%, respectively. Hence, 65% for %VC and 80% for FEV_1_/FVC were set as the cut‐off points for this study. There were 419 patients with %VC ≥ 65% (76.5%) and 328 patients with FEV_1_/FVC ≥ 80% (59.9%). The average values of %VC and FEV_1_/FVC were 79.9% and 81.2%, respectively.

### 3.3 Recovery rate of BALF

**FIGURE 3:**
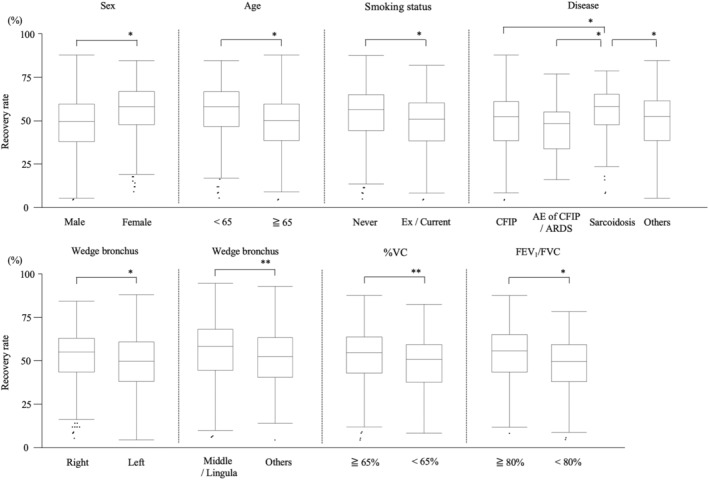
The recovery rate of bronchoalveolar lavage fluid according to sex, age, smoking status, disease, use of the bronchus for the procedure, and pulmonary function. %VC: Predicted percentage for vital capacity, AE, acute exacerbation; ARDS, acute respiratory distress syndrome; CFIP, chronic fibrotic interstitial pneumonia; FEV_1_/FVC, forced expiratory volume in 1 s divided by forced vital capacity. ^*^ *p* < 0.001, ^**^ *p* < 0.005

Figure 3 and Table 2 show the recovery rates of BALF for each parameter. The average recovery rate was 52.4% (range 9.0%–86.0%). Factors associated with higher recovery rates were female sex (female 56.2% vs. male 49.7%, *p* < 0.001), age < 65 (age < 65 [56.2%] vs. age ≥ 65 [50.0%], *p* < 0.001) and non‐smoking status (non‐smoker 55.0% vs. ex/current smoker 50.7%, *p* < 0.001). Patients with sarcoidosis had a better recovery rate than patients with CFIP without AE, AE of CFIP/ARDS and others (sarcoidosis 57.7% vs. CFIP without AE 51.0%, AE of CFIP/ARDS 48.0% and others 51.1%, *p* < 0.001). Regarding laterality and location of the bronchus, higher recovery rates were observed in the right bronchus and the middle lobe bronchus or lingula (right 53.7% vs. left 50.0%, *p* < 0.001; middle lobe or lingula 53.5% vs. others 49.2%, *p* = 0.002). Higher recovery rates were observed in the high respiratory function group than in patients with decreased respiratory function (%VC ≥ 65% [53.4%] vs. <65% [50.1%], *p* = 0.022; FEV_1_/FVC ≥ 80% [54.8%] vs. <80% [49.4%], *p* < 0.001).

**TABLE 2:** Factors affecting the recovery rate of BALF

Variables		Number of patients (*n*)	Recovery rate (%)	95%CI	*p*‐value
Average (range)		826	52.4 (9.0–86.0)		
Sex	Male	476	49.7	48.4–50.9	<0.001
	Female	350	56.2	54.7–57.7	
Age	<65 years	333	56.2	54.5–57.6	<0.001
	≥65 years	493	50.0	48.7–51.2	
Smoking status	Non‐smoker	321	55.0	53.4–56.5	<0.001
	Ex/current smoker	481	50.7	49.4–51.9	
Disease	CFIP without AE	246	51.0	49.2–52.7	<0.001
	Sarcoidosis	187	57.7	55.7–59.8	
	AE of CFIP/ARDS	36	48.0	43.4–52.7	
	Others	357	51.1	49.7–52.6	
Bronchus laterality	Right	576	53.7	52.5–54.9	<0.001
	Left	235	50.0	47.9–51.6	
Bronchus location	Middle/lingula	630	53.5	52.4–54.6	0.002
	Others	182	49.2	47.2–51.3	
%VC	≥65%	417	53.4	52.1–54.8	0.022
	<65%	129	50.1	47.7–52.6	
FEV1/FVC	≥80%	327	54.8	53.3–56.3	<0.001
	<80%	219	49.4	47.5–51.2	

Abbreviations: AE, acute exacerbation; ARDS, acute respiratory distress syndrome; BALF, bronchoalveolar lavage fluid; CFIP, chronic fibrotic interstitial pneumonia; CI, confidence interval; FEV_1_/FVC, forced expiratory volume in 1 s divided by forced vital capacity; VC, vital capacity.

### 3.4 Factors affecting recovery rate of BALF

Table 3 summarizes the results of the univariate and multivariate logistic regression analyses. There were 535 patients used for logistic regression analyses because 11 patients had a part of missing essential data. Multivariate logistic regression analysis found that male sex (odds ratio [OR]: 0.32, 95% confidence interval [CI]: 0.20–0.53, *p* < 0.001), age ≥ 65 years (OR: 0.50, 95% CI: 0.33–0.76, *p* < 0.001), use of the left bronchus (OR: 0.46, 95% CI: 0.30–0.71, *p* = 0.001), use of bronchi other than the middle lobe bronchus or lingula (OR: 0.41, 95% CI: 0.25–0.65, *p* < 0.001) and FEV_1_/FVC < 80% (OR: 0.42, 95% CI: 0.40–1.00, *p* < 0.001) were associated with lower BALF recovery rates. This model showed good discrimination, as demonstrated by a C‐statistic of 0.73 (95% CI: 0.69–0.78).

**TABLE 3:** Logistic regression analysis of the effect on the recovery rate of BALF for each parameter (*n* = 535)

Variables		Univariate logistic regression			Multivariate logistic regression		
		OR (95%CI)		*p*‐value	OR (95%CI)		*p*‐value
Sex	Male	0.37	0.25–0.54	<0.001	0.32	0.20–0.53	<0.001
	Female	REF			REF		
Age	≥65 years	0.48	0.33–0.70	<0.001	0.50	0.33–0.76	<0.001
	<65 years	REF			REF		
Smoking status	Ex/current smoker	0.64	0.45–0.93	0.018	1.25	0.77–2.02	0.360
	Non‐smoker	REF					
Disease	CFIP without AE	0.40	0.24–0.64	<0.001	0.71	0.41–1.26	0.246
	AE of CFIP/ARDS	0.34	0.11–1.09	0.070	0.65	0.18–2.32	0.510
	Others	0.38	0.23–0.63	<0.001	0.68	0.39–1.20	0.184
	Sarcoidosis	REF			REF		
Bronchus laterality	Left	0.47	0.32–0.69	<0.001	0.46	0.30–0.71	<0.001
	Right	REF			REF		
Bronchus location	Others	0.47	0.31–0.71	<0.001	0.41	0.25–0.65	<0.001
	Middle/lingula	REF			REF		
%VC	<65%	0.62	0.42–0.93	0.021	0.63	0.40–1.00	0.050
	≥65%	REF			REF		
FEV_1_/FVC	<80%	0.59	0.41–0.84	0.003	0.42	0.28–0.63	<0.001
	≥80%	REF			REF		

*Note*: The C‐statistic showed good discrimination in the multivariate logistic model (C = 0.73, 95% CI: 0.69–0.78).

Abbreviations: AE, acute exacerbation; ARDS, acute respiratory distress syndrome; BALF, bronchoalveolar lavage fluid; CFIP, chronic fibrotic interstitial pneumonia; CI, confidence interval; FEV_1_/FVC, forced expiratory volume in 1 s divided by forced vital capacity; VC, vital capacity.

### 3.5 Analysis in the CFIP group

The same analysis was performed on CFIP patients. There were 173 idiopathic interstitial pneumonias, 23 connective tissue disease‐interstitial lung disease and three chronic hypersensitivity pneumonitis patients in this group. Table 4 shows the detailed HRCT findings of the CFIP pattern. BALF was performed in the middle lobe bronchus or lingula in a total of 203 cases, and the HRCT findings of most patients showed ground glass shadow or reticulation. The recovery rates and logistic regression analyses are shown in Tables 5 and 6. Higher recovery rates were observed in females and non‐smokers, with the following parameters: use of the right bronchus (vs. the left bronchus) and FEV_1_/FVC ≥ 80% (vs. FEV1/FVC < 80%). Furthermore, male sex (OR: 0.52, 95% CI: 0.25–1.12, *p* = 0.094), use of the left bronchus (OR: 0.46, 95% CI: 0.25–0.85, *p* = 0.013), %VC < 65% (OR: 0.37, 95% CI: 0.19–0.71, *p* = 0.003) and a FEV_1_/FVC < 80% (OR: 0.51, 95% CI: 0.27–0.96, *p* = 0.037) were associated with lower BALF recovery rates. The C‐statistic for this model was 0.67 (95% CI: 0.60–0.75).

**TABLE 4:** The HRCT findings of CFIP pattern at the site of BAL.

HRCT findings	Number of patients (*n*)	
Ground glass shadow	110	44.7%
Consolidation	14	5.7%
Reticulation	201	81.7%
Traction bronchiectasis	97	39.4%
Honeycomb (single layer)	25	10.2%
Honeycomb (multiple layers)	31	12.6%
Low attenuation area without honeycomb	13	5.3%
Unclassifiable abnormal shadow	4	1.6%
Any abnormal changes	230	93.5%

Abbreviations: BAL, bronchoalveolar lavage; CFIP, chronic fibrotic interstitial pneumonia; HRCT, high‐resolution computed tomography.

**TABLE 5:** Factors affecting the recovery rate of BALF in the CFIP group

Variables		Number of patients (*n*)	Recovery rate (%)	95%CI	*p*‐value
Average (range)		214	51.1 (9.0–86.0)		
Sex	Male	144	49.4	47.1–51.8	0.022
	Female	70	54.4	50.9–57.8	
Age	<65 years	50	51.2	46.4–54.7	0.777
	≥65 years	164	50.5	48.9–53.5	
Smoking status	Non‐smoker	68	53.7	50.2–57.2	0.078
	Ex/current smoker	146	49.7	47.2–52.3	
Bronchus laterality	Right	131	52.7	50.1–55.2	0.046
	Left	83	48.5	45.4–51.7	
Bronchus location	Middle/lingula	38	50.5	45.8–55.2	0.806
	Others	176	51.2	49.0–53.4	
%VC	≥65%	144	51.4	48.9–53.8	0.661
	<65%	70	50.4	46.9–53.9	
FEV1/FVC	≥80%	150	53.3	51.0–55.7	<0.001
	<80%	64	45.7	42.2–49.2	

Abbreviations: BALF, bronchoalveolar lavage fluid; CFIP, chronic fibrotic interstitial pneumonia; CI, confidence interval; FEV_1_/FVC, forced expiratory volume in 1 s divided by forced vital capacity.; VC, vital capacity.

**TABLE 6:** Logistic regression analysis of the effect on the recovery rate of BALF for each parameter, in the CFIP group (*n* = 214)

Variables		Univariate logistic regression			Multivariate logistic regression		
		OR (95%CI)		*p*‐value	OR (95%CI)		*p*‐value
Sex	Male	0.60	0.33–1.08	0.091	0.52	0.25–1.12	0.094
	Female	REF			REF		
Age	<65 years	1.08	0.57–2.05	0.809	0.96	0.49–1.89	0.898
	≥65 years	REF			REF		
Smoking status	Ex/current smoker	0.72	0.40–1.29	0.268	0.99	0.47–2.12	0.985
	Non‐smoker	REF			REF		
Bronchus laterality	Left	0.58	0.33–1.02	0.058	0.46	0.25–0.85	0.013
	Right	REF			REF		
Bronchus location	Others	0.69	0.34–1.40	0.306	0.53	0.25–1.13	0.101
	Middle/lingula	REF			REF		
%VC	<65%	0.45	0.25–0.82	0.009	0.37	0.19–0.71	0.003
	≥65%	REF			REF		
FEV_1_/FVC	<80%	0.76	0.43–1.34	0.341	0.51	0.27–0.96	0.037
	≥80%	REF			REF		

*Note*: The C‐statistics of multivariate logistic model is 0.67 (95%CI: 0.60–0.75).

Abbreviations: BALF, bronchoalveolar lavage fluid; CFIP, chronic fibrotic interstitial pneumonia; CI, confidence interval; FEV1/FVC, forced expiratory volume in 1 s divided by forced vital capacity; VC, vital capacity.

## 4 DISCUSSION

The present study demonstrated that BALF recovery rate is associated with several clinical parameters, including sex, age, disease, the region of the bronchus used for the procedure and pulmonary function, and the potential use of these parameters as predictive factors could assist clinical decision‐making, specifically regarding whether to perform BAL in certain patients. To the best of our knowledge, this is the first study to investigate potential factors affecting the recovery rate of BALF.

It has been hypothesized that various clinical factors, mainly inherent to the patients and the BAL procedure itself, influence the recovery rate of BAL. Patient factors include age, sex, underlying respiratory disease state (especially lung disease and lung compliance) and the anatomical structure of the bronchi. On the other hand, BAL procedure factors include the expertise of the operator and assistant, as well as the difference in the diameters between the bronchoscope and target bronchus.

In our study, the BALF recovery rate was significantly lower in those over the age of 65 years compared with younger patients. Olsen et al. also published a study on 295 healthy non‐smoking volunteers, in which they reported a statistically significant association between age and the recovery rate of BALF.^11^ One possible explanation is the deterioration of lung compliance due to ageing. However, in our study, multivariate analysis showed that age ≥ 65 years was an independent factor associated with low recovery rates, regardless of respiratory function (Table 3). Another possible explanation is the atrophy of the bronchial glands and mucosa due to ageing.^14^ As a result, a slight gap could occur between the tip of the bronchoscope and the lumen of the bronchus; this could result in the leaking of physiological saline or insufficient negative pressure.

Our study showed that the recovery rate in males was lower than in females. One reason may be that males have relatively larger lung volumes than females,^15^ which could result in the retention of physiological saline. The recovery rate was better in the right bronchus when compared with the left and in the middle lobe bronchus or lingula bronchi compared with other locations. In general, BAL is performed in the middle lobe bronchus or lingula bronchi and the middle lobe bronchus rather than the lingula.^2^, ^3^, ^9^ We found no evidence that other locations were associated with a higher recovery rate. Despite being considered solely empirical knowledge, our conclusions are compatible with generally accepted findings. With a patient in the supine position, the orifice of the middle lobe bronchus and lingula are located in areas that resist gravity. The middle lobe bronchus divides into the medial and lateral bronchus, and the lingula divides into the superior and inferior bronchus; thus, the middle bronchus would be more horizontal. Therefore, gravity may facilitate the recovery of the injected saline.

In this study, the recovery rate was significantly lower in the group with a low FEV_1_/FVC ratio; this may have been attributable to a loss of lung elastic tissue and hyperinflation. In a previous study of 20 patients with chronic obstructive pulmonary disease (COPD) who underwent BAL, the FEV_1_/FVC ratio and the degree of emphysema were associated with BALF recovery rates.^12^ Smoking causes bronchiolitis, which is followed by airway remodeling. This, in turn, leads to chronic airway obstruction, which is reflected by a reduced FEV_1_/FVC. In addition, smoking damages the lung parenchyma, leading to the development of pulmonary emphysema and the loss of lung elastic tissue.^16^ Bronchiolitis and an increase in lung compliance raise the risk of bronchial collapse during the application of negative pressure during BAL, leading to a low recovery rate.^12^ On the other hand, our study indicated that BALF recovery rates were unaffected by smoking status. Previous studies have reported that the extent of emphysema may predict a lower recovery rate of BALF in COPD.^17^ Furthermore, smoking is a known cause of bronchial gland and mucosal atrophy.^17^ Our results may have been due to the inclusion of a considerable number of patients with only a mild smoking habit, and there were not many cases with imaging changes such as emphysema and/or bronchiolitis; this may have accounted for the lack of statistical significance observed.

The decrease of the %VC in the CFIP group was associated with a lower BALF recovery rate; this has not been previously reported. A low %VC reflects a decrease in lung compliance, resulting in the lack of a force to push out BALF, despite negative pressure. Furthermore, in cases of CFIP, a honeycomb pattern and cysts are observed. This anatomical change is often seen in the subpleural region^13^ and becomes wider in cases of low %VC.^18^ In this study, there were not many cases in which a honeycomb pattern and cysts were found at the site of the BAL (22.8% and 5.3%, respectively); however, abnormal findings were observed in 93.5% of the patients. These structures could trap BALF, thus leading to a lower recovery rate.

Low recovery rates of BALF may not only lead to an inaccurate diagnosis but may also lead to an increase in adverse events.^19^ In this study, we performed AUC analysis by dividing the groups by the recovery rate of 50%. Abe et al. reported that the recovery rate of BALF in patients with AE of idiopathic pulmonary fibrosis after BAL was 17%–56%.^20^ If the recovery rate is less than 50%, the frequency of complications may be higher. Our study demonstrated that use of the left bronchus during BAL and lower respiratory function led to a lower recovery rate of BALF in CFIP. Therefore, the BAL procedure should be performed with discretion in cases with a high likelihood of lower recovery rates.

There were several limitations to our study. First, this was a retrospective and single‐centre study. Patients with contraindications to bronchoscopy, severe hypoxemia and/or lower respiratory function tended to be precluded from the procedure. Therefore, this study was not likely to include patients with a severe disease state; this may have potentially resulted in a selection bias. However, AE of CFIP/ARDS patients tended to be with respiratory failure and have a low recovery rate. The causes should be discussed, including PaO_2_/FiO_2_, technique and procedure time. A prospective, multicentre study with standardized criteria for BAL would be suitable to address these concerns. Second, the recovery rate of BALF is highly dependent on the expertise of the operator. In this study, bronchoscopies were performed by pulmonologists who had over 5 years of experience. Furthermore, the recovery rate also depends on the operator's technical skills (e.g., maintenance of correct positioning during the procedure) and bronchoscopic findings. Nevertheless, both factors were difficult to evaluate and may have acted as unmeasured confounding variables. A future study may be warranted to explore the effects of these parameters.

In conclusion, the recovery rate of BALF is affected by several factors, including sex, age, disease state, use of the bronchus for the procedure and pulmonary function. These offer potential as pre‐procedural effectiveness predictors that could provide clinical guidance.

## CONFLICT OF INTEREST

The authors have no conflicts of interest to declare.

## ETHICS STATEMENT

The study was performed in accordance with the amended Declaration of Helsinki. Written informed consent for bronchoscopy was obtained from each patient. Data anonymization and privacy issues were strictly addressed, and the study protocol with an opt‐out consent method was approved by the Human Ethics Committee of Chiba University Hospital (approval number 3833).

## AUTHOR CONTRIBUTIONS

All authors contributed to the conceptual design of the survey. KS, MA, KT, KY, TI, TK, JI and JT performed BAL procedures. KS, MA, YS and YK performed the statistical analysis. All authors read and approved the final manuscript.

## Supporting information


**Table S1** Mapping of Methodology to the Design Research Phases of Intervention Development

## Data Availability

Derived data supporting the findings of this study are available from the corresponding author on request.
